# *Nursing Reports*: Annual Report Card 2020

**DOI:** 10.3390/nursrep11020020

**Published:** 2021-03-29

**Authors:** Richard Gray

**Affiliations:** School of Nursing and Midwifery, La Trobe University, Melbourne 3086, Australia; R.Gray@latrobe.edu.au

The choices authors make about where to submit their papers are complicated but are typically based on the Journal’s prestige. Prestige is generally determined using metrics based on the number of citations papers have received in a journal over a specific period. The measure that is perhaps most influencial is the Journal Citation Report, Impact Factor [1]. There are, of course, other citation-based metrics, the Google Scholar h5-index, Scimago Journal and Country Rank and SCOPUS cite score, for example, but they do not have the recognition or influence of the Impact Factor. However, although these citation metrics are calculated using a different formula, they are all essentially measuring the same thing, how often papers published in a given journal are cited in other journals. Not surprisingly, they all strongly correlate with each other [1]. Is the citation really the only metric to determine the quality and prestige of an academic journal?

In a recent editorial in the *Journal of Advanced Nursing*, I argued that there are other metrics—time papers are in review, rejection rate—that Journal editors should tell their readers about to enable them to make an more informed and rounded judgement about where to submit their research [1]. In my first editorial as editor-in-chief of *Nursing Reports*, I said that I—along with my associate editor, editor and editorial board colleagues—would publish an annual report card about how the Journal is doing and how, based on those observations, we planned to improve the Journal over the coming year [2]. This is that report.

MDPI took over publishing Nursing Reports in July 2020, and I became Editor-in-Chief in July of that year, I was invited by the publisher to take on the role, and my appointment is for two years. Consequently, this “annual” report card only covers July through December 2020.

## 1. Report Card

### 1.1. Associate Editors

*Nursing Reports* currently has four Associate Editors; two stepped down in 2020. We did not make any new associate editor appointments in 2020.

### 1.2. Editors and Assistant Editors

Eighteen editors and assistant editors work on Nursing Reports; they are employed by MDPI and work across several MDPI journals.

### 1.3. Editorial Board Membership

In total we have appointed 25 new editorial board members meaning that our board currently comprises 32 members; most identify as female (*n* = 19, 59.37%) and work primarily in Europe (*n* = 19, 59%), Australia (*n* = 6, 19%), North America (*n* = 4, 13%) and Asia (*n* = 3, 9%).

### 1.4. Submission and Publications

Between 1 July and 31 December 2020, 69 papers were submitted to *Nursing Reports* for consideration for publication. In total, we published 23 documents, of which the majority were original research (15/23, 65%) and systematic literature reviews (3/23, 14%). Additionally, we published one editorial and no letters.

### 1.5. Review Process

All papers—except editorials and letters to the editor—were subject to peer review. All reviewers acknowledged any potential conflicts of interest before reviewing the paper. At least two reviewers reviewed all papers; the average number of reviewers per paper was 2.8 (range 2–4), 13 papers had three or more reviewers. The average time from submission to first decision was 17 days (range 11–50). 

### 1.6. Time from Acceptance to Publication 

From acceptance to the paper being published online in its final form took on average 4.5 days (range 2 to 24).

### 1.7. Appeals

In 2020 none of the authors of papers that we rejected appealed our decision. 

### 1.8. Availability of Study Data

Of the 23 papers that we published in 2020, none of the authors indicated source data were available. 

### 1.9. Pre-Registration of Observational and Experimental Studies

We did not publish any randomized controlled trials in 2020. Of the 17 observational studies we published, none were registered with a WHO-approved registry. One protocol was registered with PROSPERO (or another relevant registry such as the Open Science Framework [OSF]). 

### 1.10. Reads, Downloads and Citations

In 2020 papers published in *Nursing Reports* were read and downloaded a total of 12,368 and 7502 times, respectively. Papers in the Journal were cited 53 times in 2020. More granular citation and download information can be access via this link: https://www.mdpi.com/journal/nursrep/stats (accessed on 27 March 2021). 

### 1.11. Special Issues

We have two *Nursing Reports* Special Issues that we are currently working on: “Evidence-Based Practice and Personalized Care” with Athena Patelarou as the editor and “Nursing and COVID-19” edited by Sonia Udod and Richard Gray. More information about these Special Issues can be access via this link: https://www.mdpi.com/journal/nursrep/special_issues (accessed on 27 March 2021). 

### 1.12. Indexing

*Nursing Reports* is indexed with Clarivate Analytics on the Emerging Sources Citation Index (ESCI). The ESCI index includes peer-reviewed publications of regional importance and in emerging scientific fields. Additionally, *Nursing Reports* is indexed in CINHAL (the Cumulative Index of Nursing and Allied Health Literature), the most extensive and in-depth nursing database covering 1937 to present. 

## 2. Plans for 2021

Across a range of metrics, *Nursing Reports* has done well over the past six months; a good number of papers have been published and are being read and cited by academic colleagues internationally. Submissions to the Journal have been handled in an exceptionally timely manner. That said, there is much work to do to achieve our objective of making *Nursing Reports* a leading nursing science journal by 2023. Below I wanted to set out some of our priorities and intended ways of working for 2021. 

### 2.1. Number of Papers

For 2021 we aim to publish 60 research or review papers, six editorials and multiple letters to the editor. 

### 2.2. Associate Editors

We are not currently looking to expand our team of associate editors. That said, we are committed that when such opportunities arise, we will follow an open, transparent, merit-based appointment process.

### 2.3. Editorial Board

We aspire to an editorial board that represents the broad constituency of nursing across the world. Throughout 2021 we plan to recruit 25 new editorial board members from geographic regions that are currently under-represented, that is to say Asia, Africa and the Middle East. We will primarily recruit by inviting esteemed colleagues to join, but also welcome post-doctoral nurse researchers applying to join the board by writing to me, setting out their research track record and explaining what they think they would bring to the Journal.

### 2.4. Peer Review

*Nursing Reports* currently operates a single-blind peer review system, that is to say, that the authors do not know the names of the reviewers, but reviewers know the names of authors. Whilst single-blinded review does have critics (see, for example, [3]), it is a more transparent process and as more studies are pre-registered is necessary so that reviewers can check to reconcile the registry entry with the trial manuscript (this cannot be practically done without authors identities being revealed). This is the model of peer review that we plan to continue with during 2021. Papers submitted to *Nursing Reports* are reviewed promptly, by way of comparison time from submission to a final decision in the *International Journal of Nursing Studies* was around 24 days (https://journalinsights.elsevier.com/journals/0020-7489/review_speed (accessed on 27 March 2021)). We note that many leading nursing science journals do not report handling times, making a direct comparison difficult.

The journal operates optional open peer-review: authors are given the option for all review reports and editorial decisions to be published alongside their manuscript. Authors can alter their choice for open review at any time before publication, but once the paper has been published changes will only be made at the discretion of the Publisher and Editor-in-Chief. In addition, reviewers can sign their review, i.e., identify themselves in the published review reports. To guarantee impartial refereeing, the names of referees will be revealed only if the referees agree to do so, and after a paper has been accepted for publication.

### 2.5. Acceptance to Publication

The time from a paper being accepted to publication is an important metric; many authors will have experienced frustration at having a paper accepted and then waiting months until the manuscript appears on the journal website for colleagues to read and digest. Again, comparative data are sparse, but we are confident that the acceptance to publication times for *Nursing Reports* are class leading. In 2021 we intend to maintain the timely production of papers accepted for publication.

### 2.6. Letter Writing

In leading medical journals, almost half of the documents published each year are letters. In nursing journals, exceptionally few letters to the editor are published. This is disappointing—and frankly concerning—as post-publication scrutiny of published research is an essential part of the scientific method [4]. I encourage colleagues to submit letters about papers published in *Nursing Reports* and hope to see an increase in the number we publish in 2021.

### 2.7. Registration and Reporting Guidelines (Badges)

Old or young, everyone loves getting a badge for doing something exceptionally well. There is not time or space in this editorial to go into detail about merits to pre-registration and adherence to reporting guidelines, except to say that both make the conduct and reporting of science better. Studies—be they experimental, observational or qualitative—published in *Nursing Reports* where the author has pre-registered (before the first participant is enrolled) the study will receive a “pre-registered study” badge. Authors that can demonstrate– via the submission of a correctly completed reporting guideline checklist (that can be accessed via https://www.equator-network.org/ (accessed on 27 March 2021)) will receive a “Reporting Guideline Compliant” badge.

### 2.8. Editorials

We aim to publish six editorials in 2021 that we hope will provoke debate and discussion among nurse scientists. It could be argued that in some nursing journal’s editorial authorship is confined to an “elite” few. We want to invite readers with an idea for an editorial to submit to the Editor-in-Chief a 50-word pitch that I, and at least one other editorial board member, will review. We will either commission you to draft and submit your editorial or provide feedback about how you might improve your idea. If colleagues want guidance on how to write an editorial, I might direct them to an editorial I wrote a few years back [5].

### 2.9. Impact Factor 

As I have already argued, Impact Factor is a metric of journal quality but by no means the only one. *Nursing Reports* is on a path to inclusion in the Journal Citation Report, and we have set ourselves the ambitious target of being included in the 2022 report that will be published in June 2023.

### 2.10. Special Issues

Special Issues are an excellent way of collating ideas around an important topic or theme. As a target, *Nursing Reports* aims to publish at least two Special Issues a year, each with approximately 10-15 papers. If you are interested in editing a special edition, we will welcome your proposal; further details can be identified via this link: https://www.mdpi.com/journalproposal/sendproposalspecialissue/nursrep (accessed on 27 March 2021).

### 2.11. Registered Reports

Registered Reports focus on the importance of the proposed research question and the rigour of methodology by reviewing and publishing the protocol before data collection. Journal editors then provisionally commit to publishing the study results as long as the research adhered to (or justified changes) the methods in the published protocol. Registered reports are intended to eradicate many questionable research practices, including low statistical power, selective reporting of results, and publication bias. Consistent with the principals and philosophy of open access publication, *Nursing Reports* is keen to implement Registered Reports in 2021. That said, nurse researchers have not engaged with registered reports, and there are few examples in the literature. Further, some nursing journals that were early adopters of registered reports have unfortunately decided to back away from this form of publication because of a “lack of good quality papers” [6]. Consequently, there is a need to promote and advocate this approach to publishing that arguably should be the norm and not the exception in nursing science.

### 2.12. Social Media

We launched a Nursing Reports Twitter account (@NursRep_MDPI) in early 2021, have been actively tweeting and are building followers. Please follow the journal and share our tweets.

## 3. Report Card Summary

The aim of this report card is to tell you—the reader—about how *Nursing Reports* is performing across a range of quality metrics that matter to you. By reflecting on our performance, I, in collaboration with associate editors, editors and editorial board members, have set out a program of work for 2021 that will further improve the quality of the Journal with the aim of *Nursing Reports* becoming seen as a target journal for nurse researchers to publish their best work. My colleagues and I want an open dialogue with readers and authors that engage with the Journal, and we welcome your feedback on ways in which you think the way we work and publish can be improved.
